# A Hybrid Raytracing and Deep Learning-Based Approach for Single-Site Localization of Radio Emitters in Urban Environment

**DOI:** 10.3390/s26196261

**Published:** 2026-10-02

**Authors:** Jaromir Gajewski, Jerzy Łopatka

**Affiliations:** Institute of Communications Systems, Faculty of Electronics, Military University of Technology, gen. Sylwester Kaliski Str. No. 2, 00-908 Warsaw, Poland; jerzy.lopatka@wat.edu.pl

**Keywords:** electromagnetic sensing, data-driven measurement techniques, urban localization, MUSIC, coherent sources, backward raytracing, UCA, sparse data, NLOS, non-cooperative emitters

## Abstract

Efficient passive radio monitoring in dense urban areas is obstructed by multipath propagation and NLOS conditions, causing conventional single-site localization methods to suffer from compounding geometric errors and sparse data availability. To address these limitations, this paper introduces a novel hybrid framework that fuses physics-based wave propagation geometry with data-driven machine learning. The proposed methodology utilizes an advanced, Circulant Modified MUSIC (CM-MUSIC) algorithm to reliably resolve highly correlated wave components on a 5-element Uniform Circular Arrays (UCA) antenna. The extracted directional inputs are processed via a backward raytracing engine to generate a continuous spatial likelihood map, which is then filtered by a Convolutional Neural Network (CNN) trained as a sector semantic mask. Empirical field validation using a low-cost Software Defined Radio (SDR) platform capturing analog FM transmissions demonstrated high tracking precision and robust noise immunity under severe spatial constraints, while an ideal sector-classification scenario analysis confirmed that the proposed framework closely converges to the theoretical accuracy limits of the geometric model. This framework provides a scalable, highly cost-effective, and hardware-preserving single-site solution for modern civilian radio spectrum regulatory enforcement.

## 1. Introduction

In recent years, the rapid growth of wireless communication technologies has exponentially increased the complexity of the electromagnetic spectrum, making efficient passive radio monitoring a critical necessity for both civilian regulatory authorities and security agencies [1]. The most important challenge within this domain is the accurate localization of non-cooperative emitters [2]—such as unauthorized transmitters, illegal broadcasting stations, or sources of jamming signals. Unlike conventional cooperative localization systems [3,4,5,6,7,8,9,10,11] (e.g., cellular networks or IoT tracking frameworks) where the target actively participates and shares a priori system parameters, passive localization must be executed entirely without the transmitter’s compliance or known signal specifications. Consequently, developing robust methods capable of identifying the precise physical coordinates of these unknown sources has become a main point of recent research in advanced sensor networks and electronic reconnaissance [12].

In dense urban areas, the propagation of radio frequency signals is naturally distorted by multipath effects, shadow fading, and the absence of a direct line-of-sight (LOS) path between the emitter and the receiver [3,13]. To address these Non-Line-of-Sight (NLOS) limitations, several recent single-site localization approaches have integrated map-assisted algorithms or raytracing techniques to reconstruct propagation paths by treating reflections as virtual sources [14]. However, a significant limitation of these geometric frameworks lies in their reliance on the a priori assumption that ideal Direction-of-Arrival (DOA) estimates are perfectly known [14]. In practical radio monitoring, obtaining accurate directional data from non-cooperative signals is obstructed by wave correlation and hardware imperfections. When directional estimators fail to deliver flawless angular inputs [15,16,17], conventional raytracing methods suffer from geometric errors, causing the reconstructed paths to diverge significantly from the actual source location.

To overcome the limitations of purely geometric methods in NLOS conditions, deep learning techniques—particularly radio fingerprinting—have been widely adopted for urban localization [18]. These AI-driven approaches map the unique multipath features of received signals to specific geographical coordinates. However, implementing a reliable fingerprinting system in real-world scenarios faces severe practical constraints that lead to the problem of sparse data availability. First, collecting a dense dataset of reference points across a modern city is a complex process that would normally span several months. This challenge is further enhanced by strict radio emission regulations and the high risk of disrupting existing commercial networks, which drastically narrows the legal time windows available for transmitting test signals. Second, urban environments are inherently fragmented by restricted areas, such as private properties, gated communities, and critical infrastructure zones. These inaccessible locations introduce significant “white spots” on the measurement map, creating a problem of sparse reference points where empirical data cannot be gathered. Consequently, purely data-driven neural networks often degrade in performance when forced to operate within these highly incomplete and discontinuous radio maps.

To bridge this gap, this paper introduces a novel hybrid localization framework specifically created to operate under severe NLOS conditions with non-cooperative emitters and highly sparse data. The core innovation of the proposed method lies in the fusion of radio propagation geometry and deep learning capabilities. Rather than relying on radio maps or flawless angular inputs, our approach utilizes an advanced, modified MUSIC algorithm designed to reliably resolve highly correlated wave components in real-world environments. To mitigate the structural simplifications of the 3D urban model and compensate for hardware measurement tolerances, the estimated direction is treated as a spatial uncertainty beam with a predefined ±2.5° [19] margin, which is then mapped into a spatial likelihood matrix via backward raytracing. Concurrently, a deep neural network is deployed to analyze the sparse signal features and identify the probable sector of the emitter localization, effectively filtering out geometrical noise by zeroing probabilities outside the predicted region. Finaly, the entire framework is validated using real-world field measurement samples, demonstrating that the combination of physics-based raytracing and data-driven sector filtration can accurately indicate emitter locations even when constrained by incomplete and fragmented empirical data.

The main contributions of this paper can be summarized as follows:

- Development of a correlation-robust DOA estimation approach for circular arrays: We propose a modified UCA MUSIC algorithm that effectively decorrelates co-channel signals in real-world environments. This addresses a critical gap in conventional decorrelation methods, which are typically limited to linear arrays (ULA) and suffer from front-back ambiguity.
- Introduction of a hybrid localization framework with spatial uncertainty modeling: We design a unique integration of backward raytracing and deep learning that successfully operates on highly fragmented maps and sparse data. By treating the directional input as a ±2.5° spatial uncertainty beam, the framework successfully mitigates the geometric inaccuracies caused by simplified 3D city models.
- Experimental validation using field-measured data: Unlike existing map-assisted frameworks validated solely by numerical simulations, the efficiency and noise robustness of the proposed system are proven using empirical signal samples captured in a dense, real-world urban environment under strict operational constraints.

The remainder of this paper is organized as follows: Section 2 describes the proposed materials and methods, including the mathematical formulation of the modified MUSIC algorithm and the hybrid localization framework. Section 3 presents the experimental results along with an ideal sector-classification scenario analysis comparing the proposed framework against an ideal sector identification baseline. Section 4 provides a comprehensive discussion on the limitations and practical implications of the method, while Section 5 concludes the paper.

## 2. Materials and Methods

The methodology proposed in this study is divided into two main sequential phases designed to resolve the challenges of non-cooperative emitter localization in harsh urban environments. First, a signal processing framework based on a modified high-resolution DOA estimation technique is established to mitigate the side effects of wave correlation at the antenna array of the multichannel KrakenSDR (KrakenRF Inc., Chicago, IL, USA) receiver. Second, a hybrid localization framework is introduced, which integrates the spatial likelihood fields derived from backward raytracing with deep-learning-driven sector filtration to accurately localize the emitter despite highly sparse data availability. The subsequent subsections provide a comprehensive mathematical formulation and technical description of each component of the developed system.

### 2.1. Proposed Modified MUSIC Algorithm

Accurate DOA estimation serves as the foundation for the entire localization framework, as any initial angular errors propagate during the spatial raytracing phase. This subsection details the development of a correlation robust angular estimator tailored for UCA, which are inherently suited for omnidirectional urban monitoring but highly sensitive to multipath signal correlation. To clearly explain the scientific advancement of the proposed modification, the theoretical background of the classical MUSIC algorithm and conventional decorrelation techniques are reviewed, followed by the mathematical derivation of our correlation-robust approach.

#### 2.1.1. Background and Conventional Decorrelation Methods

To establish the foundation for the proposed framework, the theoretical model of the classical MUSIC algorithm must be formulated. Consider an array consisting of *M* identical, omnidirectional antennas. Assuming *K* narrowband, co-channel wavefronts arriving on the array from distinct DOAs *θ_k_* (*k* = 1, 2, …, *K*) and *K* < *M*, the complex signal vector *x*(*t*) received by the array at any discrete time *t* can be mathematically modeled as:

*x*(*t*) = *A*(*θ*)*s*(*t*) + *n*(*t*), (1)

where *s*(*t*) = [*s*_1_(*t*), *s*_2_(*t*), …, *s_K_*(*t*)]*^T^* denotes the vector of incident source signals, *n*(*t*) represents the additive white Gaussian noise (AWGN) vector with zero mean and variance *σ*^2^, and *A*(*θ*) = [*a*(*θ*_1_), *a*(*θ*_2_), …, *a*(*θ_K_*)] is the array steering matrix composed of the geometry-dependent steering vectors *a*(*θ_k_*).

In practical applications, the spatial covariance matrix *R_xx_* of the received signal vector is estimated over *N* discrete snapshots as follows:

(2) $$R_{\mathrm{xx}}=\frac{1}{N}\sum_{t=1}^{N}x\left(t\right)x^{H}\left(t\right)=A\left(\theta \right)R_{\mathrm{ss}}A^{H}\left(\theta \right)+\;\sigma^{2}I_{M},$$

where (*∙*)*^H^* denotes the conjugate transpose operator, *I_M_* is the *M* × *M* identity matrix, and *R_ss_* = *E*{*s*(*t*)*s^H^*(*t*)} represents the source signal covariance matrix. When the incoming source signals are completely uncorrelated, the matrix *R_ss_* maintains full rank (*rank*(*R_ss_*) = *K*), rendering it non-singular. Under this condition, an eigenvalue decomposition (EVD) of the spatial covariance matrix *R_xx_* can be performed:

(3) $$R_{\mathrm{xx}}=U_{s}\Lambda_{s}U_{s}^{H}+U_{n}\Lambda_{n}U_{n}^{H},$$

where *Λ_s_* contains *K* largest eigenvalues corresponding to the signal subspace spanned by the columns of *U_s_*, while *Λ_n_* contains the remaining *M*-*K* noise eigenvalues, whose corresponding eigenvalues in *U_n_* span the orthogonal noise subspace.

Due to the fundamental property of subspace orthogonality, the steering vectors of the true incident angles are strictly orthogonal to the noise subspace, meaning that *a^H^*(*θ*)*U_n_* = 0 only when *θ* ∈ {*θ*_1_,*θ*_2_,…,*θ_K_*}. Exploiting this relationship, the classical MUSIC spatial pseudospectrum *P_MUSIC_*(*θ*) is computed by scanning the angular domain as:

(4) $$P_{\mathrm{MUSIC}}\left(\theta \right)=\;\frac{1}{a^{H}(\theta )U_{n}U_{n}^{H}a(\theta )}.$$

Consequently, when *θ* matches the true DOA of an incident wave, the denominator of Equation (4) approaches zero, producing sharp, high-resolution peaks that correspond to the estimated emitter directions [20,21].

However, the high-resolution capability of the classical MUSIC algorithm degrades severely in the presence of coherent or highly correlated signals, which are omnipresent in dense urban NLOS environments due to intense multipath propagation and reflections. Under such conditions, the incident wavefronts are delayed and scaled replicas of the same original source transmission. Mathematically, this physical phenomenon induces a strict linear dependence among the rows and columns of the source covariance matrix *R_ss_*, causing it to lose its full rank properties such that:

*rank*(*R_ss_*) < *K*. (5)

As a consequence of this rank deficiency, the signal eigenvalues in Equation (3) leak into noise subspace, and the steering vectors associated with the true DOAs are no longer orthogonal to columns of *U_n_*. Therefore, the denominator of the spatial pseudospectrum in Equation (4) fails to approach zero at the actual incident angles, resulting in merged, significantly flattened, or completely omitted peaks in *P_MUSIC_*(*θ*).

To restore the rank of the signal covariance matrix, the spatial smoothing technique is conventionally employed in array processing. This approach artificially decorrelates coherent signals by dividing the overall antenna array into multiple overlapping subarrays and averaging their respective local covariance matrices [20,22]. While spatial smoothing is mathematically capable of restoring *R_ss_* to full rank, it introduces a severe hardware penalty. By forcing the algorithm to operate on smaller spatial segments rather than the entire array simultaneously, the technique inherently reduces the effective physical aperture of the antenna system. This reduction in effective physical aperture compromises the angular resolution of the sensor grid and reduces the maximum number of co-channel emitter sources that the system can simultaneously resolve, rendering it highly inefficient for resource-limited radio monitoring stations.

To overcome the physical aperture loss associated with spatial smoothing, alternative hardware-preserving decorrelation techniques have been proposed. A prominent approach is the Modified MUSIC (MMUSIC) algorithm [16], which attempts to restore the rank of the covariance matrix by generating a forward-backward averaged matrix *R_MOD_* defined as:

(6) $$R_{\mathrm{MOD}}=\frac{R_{\mathrm{xx}}+J_{M}R_{\mathrm{xx}}^{*}J_{M}}{2},$$

where $R_{\mathrm{xx}}^{*}$ denotes the complex conjugate of the estimated covariance matrix, and *J_M_* represents an *M* × *M* exchange matrix featuring ones along its anti-diagonal and zeros elsewhere. While this transformation successfully restores the matrix rank without reducing the array dimensions, its mathematical validity strictly relies on the assumption that the array steering matrix *A*(*θ*) possesses a conjugate-symmetric Vandermonde structure. Consequently, the application of Equation (6) is fundamentally restricted to linear antenna arrays.

Further advancements, such as the multi-subspace algorithm proposed by A. Borkar [17], attempt to enhance low-SNR resolution by transforming the original covariance matrix *R_xx_* into a Hermite–Toeplitz matrix *R_T_* through diagonal averaging. Instead of relying purely on the orthogonal noise subspace, this approach calculates the singular value decomposition (SVD) of both the forward–backward averaged *R_MOD_* and a rank-reduced version of *R_T_*. The resulting signal and noise eigenvectors are subsequently averaged to construct a modified multi-space spatial pseudospectrum *P_new_*(*θ*), defined as:

(7) $$P_{\mathrm{new}}\left(\theta \right)=\frac{a^{H}\left(\theta \right)[V_{s}\Lambda^{-}V_{s}^{H}]a(\theta )}{a^{H}{(\theta )V}_{n}V_{n}^{H}a(\theta )},$$

where *V_s_* and *V_n_* represent the averaged signal and noise subspaces, respectively, and *Λ^-^* denotes the generalized inverse of the eigenvalue matrix. By incorporating the signal subspace into the numerator, this technique sharpens the spectral peaks for coherent sources.

Although both the MMUSIC and the multi-subspace Toeplitz–SVD frameworks yield high angular resolution for linear grids, they encounter a critical geometric barrier when applied to UCAs. Due to the inherent angular dependencies and trigonometric exponents in the steering vectors of architectures, the spatial covariance matrix lacks both spatial shift invariance and Vandermonde properties. Consequently, applying ULA-specific transformations such as standard exchange-matrix multiplication, diagonal averaging, or linear subspace blending change the spatial phase information. In UCA configurations, these conventional approaches lead to severe estimation bias or a complete breakdown of the directional estimator, highlighting the need for a dedicated, geometry-aware decorrelation method.

#### 2.1.2. Circulant Modified MUSIC Algorithm

To effectively resolve coherent wavefronts without degrading the physical aperture of the antenna array, this paper introduces a novel Circulant Modified MUSIC (CM-MUSIC) algorithm tailored specifically for UCAs. The core innovation of the proposed method lies in the circular spatial averaging of the covariance matrix across all radial axes of symmetry inherent to the circular geometry. Let *R_xx_* be the conventional spatial covariance matrix estimated using Equation (2). To perform omnidirectional decorrelation, a set of cyclic exchange matrices is generated. First, a baseline anti-diagonal exchange matrix *J_M_* is defined. Subsequently, for each antenna element *m* = 0, 1, …, *M*–1, a unique rotation-reflection matrix *J_M_* is constructed by applying a vertical cyclic shift to the baseline matrix:

*J_m_* = *circshift*(*J_M_*,*m*,1). (8)

Geometrically, each *J_M_* matrix represents a spatial reflection operator corresponding to a specific axis of symmetry passing through the circular grid. The omnidirectionally decorrelated covariance matrix *R_CM_* is then formulated by accumulating these cyclic mirror transformations and combining them with the original covariance estimate:

(9) $$R_{\mathrm{CM}}=R_{\mathrm{xx}}+\frac{1}{M}\sum_{m=0}^{M-1}J_{m}R_{\mathrm{xx}}^{*}J_{m},$$

where $R_{\mathrm{xx}}^{*}$ denotes the complex conjugate matrix. By averaging the spatial phase dependencies over all localized reflection planes, Equation (9) successfully restores the rank of the source covariance matrix for coherent multipath signals coming from any azimuth direction within a 360-degree range, while preserving the full dimensional aperture of the array.

Following the construction of the circulant modified matrix, an eigenvalue decomposition is executed:

*R_CM_* = *VDV^H^*, (10)

where *D* = *diag*(*λ*_1_, *λ*_2_, …, *λ_M_*) represents the diagonal matrix of sorted eigenvalues, and *V* contains the corresponding eigenvectors. To automatically determine the number of correlated signal paths without relying on stiff information criteria, a dedicated Energy Criterion is introduced. The total received signal power *P_total_* is calculated as the trace of eigenvalue matrix:

(11) $$P_{\mathrm{total}}=\sum_{i=1}^{M}\lambda_{i}.$$

Each individual eigenvalue is then evaluated to determine its percentage contribution *ρ_i_* to the overall energy, such that *ρ_i_* = (*λ_i_*/*P_total_*) × 100%. Under the physical assumption that the dominant signal components contain at least 95% of the total captured power, the dimension of the signal subspace is adaptively selected by finding the minimum number of eigenvalues required to satisfy the following energy threshold:

(12) $$\sum_{i=1}^{M}\rho_{i}\geq 95\%.$$

Based on the dynamically estimated signal subspace, the eigenvector matrix *V* is partitioned into signal subspace *V_s_* and the orthogonal noise subspace *V_n_*.

To further sharpen the resolution peaks in dense multipath environments, the signal subspace and its corresponding energy eigenvalues are integrated into the directional scanning process. Utilizing the partitioned subspaces, a signal-energy reconstruction matrix is generated and inverted using Moore–Penrose generalized pseudoinverse [23]:

(13) $$R_{\mathrm{sa}}^{-}={\left(V_{s}D_{s}V_{s}^{H}\right)}^{†},$$

where *D_s_* is a truncated diagonal matrix containing only dominant signal eigenvalues.

During the spatial scanning phase across 360-degree azimuth domain, the geometry-specific steering vector *a*(*θ*) for the UCA is defined according to the empirical field phase convention as:

(14) $$a\left(\theta \right)={\left[e^{-\mathrm{jkrcos}\left(\theta +\gamma_{0}\right)},e^{-\mathrm{jkrcos}\left(\theta +\gamma_{1}\right)},\;\ldots ,\;e^{-\mathrm{jkrcos}\left(\theta +\gamma_{M-1}\right)}\right]}^{T},$$

where *k* = 2*πf*/*c* is the wavenumber, *f* denotes the operating frequency of the incident wavefront, *c* is the speed of light in free space, *r* denotes the physical radius of the circular array in meters, and *γ_m_* = 2*πm*/*M* represents the spatial angular position of the *m*-th antenna element. Finally, the proposed circular multi-subspace spatial pseudospectrum *P_CM-MUSIC_* is computed as the ratio of the signal-space projection to the noise-space orthogonal projection:

(15) $$P_{\mathrm{CM}\text{-}\mathrm{MUSIC}}\left(\theta \right)=\;\frac{a^{H}(\theta )R_{\mathrm{sa}}^{-}a(\theta )}{a^{H}(\theta )(V_{n}V_{n}^{H})a(\theta )}.$$

Upon evaluating Equation (15), the mathematical interaction between the energy-weighted numerator and the noise-orthogonal denominator suppresses the spectral baseline while narrowing the beamwidth of the directional peaks, ensuring robust and highly accurate angle-of-arrival estimates for the subsequent spatial raytracing stages.

#### 2.1.3. Preliminary Simulation and Performance Comparison

To evaluate the results of the proposed CM-MUSIC algorithm prior to its integration into the hybrid localization method, numerical simulations were conducted. The simulation parameters were selected to reflect the physical hardware constraints and operational environment of the field measurements described in later sections. The sensor grid was modelled as a 5-element UCA with a radius of *r* = 26.3 cm. The operating frequency was set to *f* = 446.15625 MHz, corresponding to the standard civilian PMR band, thereby matching the emissions of the commercial handheld transceivers utilized in the real-world validation. The received signals were frequency-modulated (FM) with a 1 kHz sinusoidal tone. To replicate low-power urban conditions, the SNR was fixed at 10 dB, and the observation window was restricted to duration *N* = 100,000 discrete snapshots. Given the 2.4 MHz sampling rate of the target receiver architecture, this snapshot volume represents an instantaneous operational capture window. All simulation parameters were listed in Table 1.

Using this unified simulation setup, the proposed CM-MUSIC approach was benchmarked against three baseline algorithms: the classical MUSIC algorithm, the MMUSIC algorithm, and the multi-subspace approach proposed by Borkar. The comparative evaluation was executed across three propagation scenarios.

##### Scenario 1: Single-Source Condition

The first simulation establishes a baseline performance bound under ideal conditions with a single incident wavefront arriving from an azimuth of *θ* = 35°.

As demonstrated in Figure 1, under these conditions, all four evaluated algorithms successfully detect the true DOA, producing distinct spectral maxima at 35°.

The classical MUSIC and MMUSIC algorithms demonstrate the highest dynamic range, maintaining a well-suppressed noise floor of approximately −65 dB. The proposed CM-MUSIC algorithm also properly identifies the correct angle; however, it exhibits a slightly elevated noise floor compared to classical MUSIC, which is a direct mathematical consequence of the circular phase-averaging process.

Despite correctly identifying the primary source, the reference methods reveal geometric vulnerabilities when forced to operate on a circular grid. Specifically, both the MMUSIC and the multi-subspace Borkar’s method generate an identical, full-amplitude false mirror projection in the negative angular domain near −145°. This artifact confirms that conventional linear-array transformations fail on non-Vandermonde steering matrices, introducing geometric ambiguity even in ideal single-source scenarios. Furthermore, the Borkar’s approach exhibits severely widened beamwidths, an elevated baseline noise floor around −15 dB, and prominent side-lobe ripples across the entire azimuth spectrum.

##### Scenario 2: Two Coherent Signals

To simulate a typical urban multipath environment where the direct wave is accompanied by NLOS reflection, a dual-source scenario was implemented with two completely coherent wavefronts striking the array from *θ*_1_ = 35° and *θ*_2_ = 100°. Simulation results are presented in Figure 2.

Under these correlated conditions, the classical MUSIC algorithm completely collapses due to rank deficiency of the source covariance matrix; it fails to produce distinct peaks for either angle, instead yielding a singular, highly distorted flat maximum centered around 70°. Both the MMUSIC and Borkars method suffer from geometric deformation, producing a set of false spectral maxima that entirely mask the true emitter positions. In contrast, the proposed CM-MUSIC algorithm successfully preserved its high-resolution capabilities, yielding two dominant, distinct spectral peaks precisely at 35° and 100°.

##### Scenario 3: Four-Path Correlated Environment

To test the absolute physical limits of the 5-element array, an extreme urban scattering scenario was modelled with four highly correlated multi-path components arriving simultaneously from *θ*_1_ = −110°, *θ*_2_ = −45°, *θ*_3_ = 15°, and *θ*_4_ = 80°. Simulation results are presented in Figure 3.

Because the number of incident wavefronts approaches the fundamental spatial degree of freedom limit of the sensor array, the comparative baseline methods experience a total breakdown, filling the angular domain with chaotic, high-amplitude noise fluctuations. The proposed CM-MUSIC algorithm, however, retains the mathematical capacity to resolve all four distinct incident angles, aligning its primary spectral peaks with the true DOAs.

##### Subspace Leakage

As observed in both the dual-source and four-source coherent scenarios, the pseudospectrum of the proposed CM-MUSIC method exhibits secondary, false maxima alongside the true target peaks. Mathematically, this behaviour is a direct consequence of the spatial averaging and rank-restoration process executed on a highly resource-constrained 5-element UCA. During the mathematical reconstruction of the covariance matrix, a minor portion of the correlated signal energy inevitably leaks to the noise subspace, slightly distorting the orthogonal projections and generating these spectral artifacts.

Because the amplitude of these false projections can occasionally approach the magnitude of the valid multipath peaks, it is impossible to establish a static threshold for automated DOA extraction within the signal processing stage alone. This crucial limitation directly justifies the necessity of the comprehensive hybrid architecture proposed in this paper.

Nevertheless, the developed CM-MUSIC algorithm appears to be the only existing solution for restoring the covariance matrix’s rank for UCA arrays under given operational assumptions. Other existing solutions that do not decrease the antenna array aperture require either a priori knowledge about the emitter or unrealistic applicational assumptions (like Davies transformation). For this reason it is impossible to compare CM-MUSIC with other methods dedicated for UCA. This creates a need for further research on this algorithm, which is the subject of our subsequent article, which is currently under preparation.

### 2.2. Hybrid Localization Framework

The methodology proposed in this study introduces a hybrid localization framework that seamlessly integrates physics-based radio propagation modelling with data-driven machine learning. This dual-path architecture is specifically engineered to overcome the geometric inaccuracies caused by simplified 3D city maps and to eliminate the false spatial peaks resulting from subspace leakage in the directional estimator. The comprehensive operational workflow of the developed localization framework is structurally mapped as a functional block diagram in Figure 4.

As illustrated in the pipeline, following the initial data acquisition and CM-MUSIC angular estimation, the system independently executes two parallel processing tracks, a geometric raytracing routine and a neural-network-based sector identification classifier, before executing a joint spatial data fusion to isolate the exact coordinates of the non-cooperative transmitter.

#### 2.2.1. Empirical Field Measurement Campaign and Data Augmentation

To compile a robust dataset reflecting genuine propagation physics and hardware behavior, a comprehensive empirical measurement campaign was conducted within the targeted dense urban area. The complete spatial layout of this field experiment is illustrated in Figure 5.

The receiver system involving a 5-element UCA with a radius of r = 26.3 cm was structurally integrated onto an aluminum platform and anchored to the roof of a test vehicle. Throughout the entire measurement campaign, this vehicle remained completely stationary at a single, fixed base position shown in Figure 5. A photograph of the actual experimental vehicle setup and the integrated antenna grid is presented in Figure 6.

The non-cooperative target transmitter utilized in this campaign was a commercial handheld Baofeng transceiver operating at a carrier frequency of *f* = 446.15625 MHz within the standard civilian PMR band [24]. Crucially, to reflect a real-world operational environment, the signal was frequency-modulated using live human speech as the baseband informational signal, rather than an idealized, deterministic synthetic tone. This transmitter was sequentially deployed across eight distinct urban sites (shown in Figure 5). At each specific site, a series of four independent signal captures was recorded. Each individual transmission snapshot lasted for 5 s, yielding a uniform and representative volume of 12 million discrete, complex samples per channel when digitized via the 5-channel coherent architecture of the KrakenSDR platform operating at a 2.4 MHz sampling rate [25].

To protect the subsequent machine learning stage against fluctuating environmental noise floors, fading effects, and hardware measurement tolerances, a comprehensive data augmentation protocol was executed offline. Each of the raw empirical signal captures was replicated and synthetically expanded by injecting AWGN across a diverse operational dynamic range spanning from 10 dB to 30 dB SNR. Through this multi-level noise injection routine, each real-world signal measurement was converted into 100 unique signal profiles (1 baseline empirical capture and 99 noise-augmented variants). Consequently, the overall training dataset was expanded to a total of 3200 distinct spectral training realizations (8 locations × 4 captures × 100 variants), ensuring that the network learns to isolate invariant directional features that remain stable even under severe channel degradation.

#### 2.2.2. Deep Learning Sector Classifier

To achieve spatial filtering and isolate the geometric region of the non-cooperative transmitter, a deep Convolutional Neural Network (CNN) was developed and implemented utilizing the MATLAB Deep Learning Toolbox version 24.1. Instead of feeding the entire continuous 360 degree CM-MUSIC pseudospectrum into the network, which would introduce destructive noise floor fluctuations and increase computational overhead, a discrete feature extraction layer was designed based on the extracted spectral peaks.

The input to the neural network is formatted as a fixed-size, three-dimensional matrix with dimensions of [2 × 765 × 1], where the third dimension represents a single feature channel. This layout is structurally organized as follows:

- Row 1 (Angular Components): Contains the discrete azimuth angles corresponding to the extracted local maxima. To compensate for hardware tolerances and the structural simplifications of the 3D urban model, each detected peak is expanded into a spatial beam spanning ± 2.5° (yielding a total angle width of 5°). Within this uncertainty angle, angular components are sampled with a tight discrete step of 0.1°, resulting in exactly 51 angular elements per resolved peak.
- Row 2 (Amplitude Components): Contains the power magnitudes corresponding to each angular element in Row 1. Instead of tracking the fading baseline of the pseudospectrum, the peak amplitude of the original maximum is uniformly duplicated across all 51 elements of its respective uncertainty beam. This configuration ensures that the network evaluates the entire spatial beam with equal weight, enhancing its capacity to discover underlying spatial correlations across different noise realizations.

To accommodate extreme multipath scattering environments while maintaining a unified network architecture, the input matrix is dimensioned to support a maximum of 15 simultaneous directional beams. In scenarios where the CM-MUSIC algorithm extracts fewer than 15 maxima, the remaining columns of the [2 × 765] matrix contain zeros. This structural standardization stabilizes the input dimension across the entire dataset without altering the physical information of the resolved paths.

The internal architecture of the proposed CNN is tailored to scan both the directional angles and their coupled power features simultaneously. The layout of the processing layers is structured as follows:

1. Image Input Layer: Accepts the raw [2 × 765 × 1] feature matrix without applying external normalization routines.
2. 2D Convolutional Layer: Deploys 128 independent filter kernels with a specialized receptive field of [2 × 5]. This rectangular kernel topology is highly critical as it spans both rows simultaneously, forcing the network to process the angle and its associated amplitude as a single, unified physical feature. The convolution utilizes identical padding to preserve the spatial borders.
3. Batch Normalization Layer: Stabilizes the internal covariate shift across the augmented noise spectrum.
4. ReLU Activation Layer: Introduces non-linearity to extract complex multipath profiles.
5. Flatten Layer: Unrolls the multi-channel feature maps into 1D vector to transition from spatial feature extraction to dense decision-making layers.
6. Fully Connected Decision Cascade: A multi-stage dense network topology designed to hierarchically reduce the data dimension. The network cascades from a dense layer of 4096 hidden units down to 64 units. To prevent overfitting on false spectral artifacts, every hidden layer is supported by a rectified linear unit (ReLU) activation and highly aggressive dropout layer, scaled down sequentially from 0.6 to 0.3. Optimization experiments revealed that skipping standard intermediate power-of-two dimensions stabilized convergence.
7. Output and Classification Layers: The final decision stage comprises a fully connected layer with 8 outputs, corresponding to the eight predefined urban surveillance sectors. A Softmax layer converts the raw output scores into a categorical probability distribution, which is finalized by a standard classification layer.

The exact architectural sequence and structural parameters of the filtering network are summarized in Table 2.

The optimization and network training phase was executed within a high-performance hardware environment utilizing dedicated GPU acceleration to ensure rapid and stable convergence. The network parameter tuning was performed using the adaptive moment estimation (Adam) optimizer, which is robust against sparse data architectures and noisy environments. To enforce high generalized performance and mitigate the effects of artificial noise injection from the augmentation stage, the initial learning rate was fixed at a highly conservative value of α = 10^−5^.

Furthermore, a rigorous L2 regularization factor of λ_L2_ = 0.002 was incorporated directly into the loss function. This unusually high regularization penalty suppresses excessively large weight profiles, forcing the network to disregard noise fluctuations and focus entirely on structural angular dependencies. The complete dataset of 3200 augmented spectral profiles was processed in optimized chunks with a mini-batch size of 128 samples. To break any localized data dependencies and artificial temporal ordering resulting from the batch acquisition campaign, the training sequence shuffled data every single epoch. The maximum training horizon was constrained to 200 epochs, providing an optimal training volume that prevents overfitting while guaranteeing the complete stabilization of the categorical cross-entropy loss function.

#### 2.2.3. Backward Raytracing and Spatial Probability Mapping

To evaluate the overall generalized localization performance of the complete hybrid architecture under blind-test conditions, an independent validation dataset was compiled immediately following the neural network training phase. This secondary verification routine involved executing a completely new, separate field measurement session. A single signal capture was recorded at each of eight transmitter coordinates under identical hardware parameters. These raw test profiles were subsequently expanded using exactly the same data augmentation method, producing an isolated validation collection of 800 unique spectral profiles that remained entirely unknown for the deep learning optimization loops.

For every directional component extracted by the CM-MUSIC algorithm during the validation session, physics-based backward raytracing was deployed to simulate wave propagation mechanics through the physical environment. The surveillance parameter under analysis includes an operational urban topography measuring 500 m by 130 m. To achieve an optimal balance between wave modelling accuracy and computational processing velocity, the deterministic raytracing engine tracks the physical interaction boundaries, modelling reflections of the simplified cuboid buildings, taking into account up to a maximum of 7 successive wave reflections. This limit was introduced because the signal energy is dissipated after each reflection, and a larger number of reflections (despite the subspace method’s high resistance to low SNR values) would prevent the signal from being detected correctly at the receiving point and it would only increase the computational requirements of the whole framework.

The core computational task within this track involves converting the discrete geometric ray trajectories into a continuous spatial likelihood field. This transformation is executed via a sequential four-stage signal-to-spatial processing pipeline:

1. Ray Path Integration and Beam Expansion: Instead of evaluating isolated linear paths for each discrete azimuth peak derived from CM-MUSIC, the spectrum is expanded into a spatial uncertainty beam covering a total arc width of 5° (± 2.5° around the peak center). Within this angular envelope, individual ray trajectories are back-propagated into a 2D grid matrix from the fixed receiver coordinates, utilizing an angular sampling interval of 0.1°.
2. Aperture Dilatation: Because idealized, infinitely thin ray boundaries do not exist in wave channels, a dilatation operator is applied to the integrated ray segments. The spatial paths are widened, which effectively thickens the linear paths, converting them into continuous volumetric propagation corridors.
3. Gaussian Energy Dispersion Filtering: To model the natural physical dispersion of electromagnetic energy across the multipath channel and to mathematically compensate for the structural simplifications of the cuboid urban buildings, the dilated propagation corridors are processed via a two-dimensional low-pass Gaussian filter. The spatial convolution with the Gaussian kernel blurs the structural edges of the beams, shifting the likelihood density to follow a bell-shaped decay curve relative to the central propagation axis.
4. Spatial Accumulation and Linear Normalization: The blurred likelihood densities derived from each independent directional peak are sequentially accumulated within a global spatial matrix. In geographical areas where multiple independent ray paths cross and intersect, the overlapping likelihood densities combine constructively. Finally, to ensure strict numerical consistency, the entire spatial field is linearly normalized, binding the absolute global maximum value to exactly 1. An illustrative example of the resulting continuous spatial likelihood heatmap generated via this method is presented in Figure 7.

#### 2.2.4. Sector Filtration and Centroid Localization

The final stage of the hybrid framework executes a spatial data fusion by combining the macro-scale sector classification from the CNN with a micro-scale continuous spatial likelihood field derived from the backward raytracing. This integration is designed to eliminate false spatial intersections and peripheral multipath noise, indicating the specific sub-region containing a non-cooperative transmitter.

The operation begins by adopting the output from the trained CNN, which identifies the most possible urban sector for given CM-MUSIC pseudospectrum peaks. Utilizing the spatial coordinate boundaries of this selected region, a dynamic two-dimensional binary mask is generated. This mask acts as a hard spatial filter across the global grid: all calculated likelihood density values falling outside the predicted sector perimeter are instantly zeroized. Immediately following this masking operation, the remaining likelihood field enclosed within the isolated sector undergoes a localized min–max normalization. This local rescaling bounds the internal density values strictly between 0 and 1, effectively sharpening and amplifying the local likelihood distribution even in sectors where the original values were heavily attenuated. An illustrative example of the resulting sector-masked and normalized spatial likelihood distribution is presented in Figure 8.

Following the local normalization, the precise geographical coordinates of the emitter are estimated. Instead of relying on a simple peak-detection threshold, which remains vulnerable to local fluctuations, the framework treats the normalized matrix as a continuous mass distribution. The target coordinates (*X*,*Y*) are mathematically computed by calculating the centroid of the likelihood field. This spatial estimation is governed by the following weighted discrete formulations:

(16) $$X=\;\frac{\sum_{i=1}^{R}\sum_{j=1}^{C}x_{j}P(i,j)}{\sum_{i=1}^{R}\sum_{j=1}^{C}P(i,j)},$$

(17) $$Y=\frac{\sum_{i=1}^{R}\sum_{j=1}^{C}y_{i}P(i,j)}{\sum_{i=1}^{R}\sum_{j=1}^{C}P(i,j)},$$

where *P*(*i*,*j*) represents the likelihood density value at the grid row index *i* and column index *j*, while *R* and *C* denote the total vertical and horizontal dimensional limits of the spatial monitoring matrix.

## 3. Results

The efficiency of the architecture is validated utilizing the independent blind-test dataset consisting of 800 noise-augmented spectral realizations derived from 8 independent physical field recordings captured across the urban measurement nodes. This structure allows for a dual-aspect evaluation: while the 8 separate physical sessions guarantee that the model is tested on entirely unseen spatial channels and live speech profiles, the 100 noise variants per location provide the necessary statistical volume to rigorously evaluate the system’s robustness against fluctuating SNR levels. To verify the developed framework, the localization metrics were benchmarked by direct comparison of the complete system performance against a standalone physics-based raytracing baseline. Furthermore, the operational limits of the method are characterized by assessing the inner classification dynamics of the deep learning sector engine.

### 3.1. Hybrid Localization Accuracy and Statistical Bound Analysis

The primary metric utilized to quantify the accuracy of the proposed framework is the linear localization error, defined as the distance between the empirically calculated centroid and the actual coordinates of the transmitter. To establish a statistical baseline, the continuous distribution of the linear error across all 800 evaluation indexes is sorted in descending order and structurally mapped against the conventional raytracing approach. This comparative performance boundary is illustrated in Figure 9.

As demonstrated by the sorted error profiles in Figure 9, the standalone backward raytracing approach (represented by the blue curve) experiences a systemic performance degradation under harsh NLOS urban propagation with sparse data. Without sector filtering, the baseline geometric engine is highly susceptible to multi-peak ambiguities and false wave intersections caused by CM-MUSIC subspace leakage, yielding a consistently high linear displacement that spans from 80 m to over 250 m across the entire evaluation sequence.

In contrast, the proposed framework (represented by the red curve) achieves a reduction in geometric error. For a significant majority of the evaluation, the linear error drops rapidly, stabilizing at a tight operational margin below 15 m. Under these optimized propagation conditions, the system demonstrates exceptional accuracy, yielding a minimum recorded localization error of just 2.05 m. This performance is further confirmed by the global sample median, which stabilizes at a value of 9.22 m.

The global mean localization error for the hybrid framework is computed at 55.52 m. As evidenced by the initial trend of the red curve, this mean value is structurally skewed by a finite cluster of high-magnitude error peaks, which correspond to specific blind-test realizations where the deep learning classifier misidentified the propagation paths. To further decouple these geometric failures from the typical operational precision of the framework, a non-parametric statistical analysis was executed on the error distribution. The resulting boxplot decomposition is illustrated in Figure 10.

The non-parametric statistical breakdown illustrated in Figure 10 further highlights the high operational precision of the proposed hybrid architecture, while clearly isolating the influence of the systemic classification failures. The lower boundary of the interquartile range (IQR), representing the 25th percentile of the linear error, evaluates to a value of 6.96 m, while the upper boundary (75th percentile) stabilizes at 44.75 m. Consequently, the entire height of the statistical box, denoting the interquartile range over which 50% of all samples are distributed, spans a tight distance of just 37.79 m.

The lower whisker of the distribution terminates at 2.05 m, matching the absolute minimum recorded error of the framework. More importantly, the upper whisker extends to 100.45 m, proving that 88.75% of the entire blind-test ensemble falls well within the hundred-meter threshold, despite intense urban multipath reflections and NLOS propagation conditions.

The analysis detected 90 statistical outliers, representing an outlier concentration of 11.25% across the 800 evaluation realizations. These anomalous points are represented as distinct red crosses spanning up to an absolute maximum recorded localization error of 410.68 m. These high-magnitude outliers are not indicative of a breakdown in the backward raytracing. Instead, they represent severe tracking anomalies where the deep learning module misidentified the transmitter sector. When CNN commits an outright classification error, the resulting spatial mask completely suppresses the valid physical wave propagation corridors, forcing the centroid calculation to operate exclusively on false multipath intersections located within the wrong sector.

To evaluate the core driver behind these spatial outliers and quantify the operational reliability of the spatial masking component, it is necessary to examine the inner classification performance of the neural network layer independently.

### 3.2. Deep Learning Sector Classification Performance Analysis

To evaluate the operational mechanics behind the calculated spatial outliers, the standalone classification capabilities of the deep learning module were analyzed utilizing the independent validation dataset. The global classification accuracy achieved by the CNN across all 800 test profiles evaluates to 62.7%, with a corresponding global error rate of 37.3%. While a 62.7% accuracy profile highlights the severe difficulty of performing non-cooperative tracking of FM-modulated NLOS channels, a deeper inspection of the internal class distributions reveals distinct physical and geometric behaviors. The resulting validation matrix is illustrated as a normalized confusion plot in Figure 11.

A critical insight into the system’s resilience can be observed by analyzing the classification failures of Sector 4. Although the direct sensitivity for Sector 4 evaluates 0%, the network systematically misclassifies these specific wavefront realizations, assigning 94% samples to Sector 1 as the output class. Because those sectors are adjacent, their respective electromagnetic reflection and scattering boundaries are nearly identical. From a localization framework standpoint, this adjacent-class misclassification is non-destructive, since the geometric backward raytracing engine continues to operate within the correct local macro-region, and the subsequent centroid calculation naturally positions the emitter localization near the boundary of the zones, preventing severe linear localization errors.

Conversely, the confusion matrix exposes the precise mathematical driver behind 90 statistical outliers and the maximum 410.68 m localization error documented in Section 3.1. Systemic tracking failures occur when the network misclassifies profiles across the boundaries of the primary macro-regions (Sectors 5–8 being projected into Sectors 1–4). The primary catalyst for these extreme errors is observed when the active transmitter is positioned in Sector 8 (Target Class = 8), where the CNN incorrectly selects Sector 1 as the output class in 37 instances. Similarly, significant cross-macro-region errors occur when the active emitter is deployed in Sector 5 or Sector 7, resulting in 20 and 15 false classifications into Sector 2.

The green cells along the diagonal on Figure 11 represent correctly classified samples, while the pink off-diagonal cells indicate misclassifications. The rightmost column shows the precision (top, green) and false (bottom, red) for each output class. The bottom row displays the recall (top, green) and false negative rate (bottom, red) for each target class. The bottom-right cell (62.7%) indicates the overall accuracy of the model.

When the CNN commits these specific macro-region classification errors, the resulting binary mask completely zeroes out the valid physical propagation corridors in the correct regions. This forces the centroid to compute the coordinates on a completely mismatched portion of the map, shifting the calculated emitter centroid away from the real coordinates and directly generating the high-magnitude linear displacements observed in the top 11.25% of the sorted error curve.

### 3.3. Ideal Sector-Classification Scenario

To isolate the performance limitations of the physical geometry engine from the classification dynamics of the deep learning module, an ablation analysis was executed by simulating a theoretical scenario. In this operational sequence, the neural network is assumed to possess ideal, flawless classification capabilities across all evaluation points. By eliminating macro-region masking errors, this analysis characterizes the baseline precision of the core CM-MUSIC directional estimator combined with the backward raytracing and centroid extraction engine. The sorted linear error distribution for this ideal CNN performance boundary is illustrated in Figure 12.

As demonstrated by the performance bound in Figure 12, the systemic elimination of sector misclassifications completely eliminates the high-magnitude localization anomalies described in previous sections. The global mean localization error drops to just 9.3 m, while the absolute maximum recorded localization error is constrained to 34.29 m. For a definitive majority of 800 validation instances, the linear error stabilizes below the 10 m threshold, converging towards single-digit metrics at the tail of the distribution. To extract the exact non-parametric statistical metrics governing this pure geometric framework, the error distribution was decomposed into a boxplot, illustrated in Figure 13.

The statistical breakdown of the ideal performance highlights an exceptional concentration of high-accuracy estimations. The lower boundary of the box evaluates to 5.11 m, while the upper boundary stabilizes at 9.63 m. Consequently, the IQR spans a remarkably tight distance of only 4.52 m.

The lower whisker terminates at 2.05 m, while the upper whisker extends to a value of 16.25 m. This implies that 92.25% of all localization attempts achieve a precision better than 16.3 m when the correct macro-region mask is deployed.

The mathematical algorithm identified 62 statistical outliers, representing a minor concentration of 7.75% across the experimental ensemble. These outliers form a localized vertical cluster extending up to a maximum bound of 34.29 m. From a wave propagation perspective, these remaining low-magnitude outliers do not stem from signal processing deficiencies of the structural abstractions implemented within the 3D urban model, where complex architectural geometries were simplified into cuboids.

### 3.4. Computational Complexity and Real-Time Feasibility

To evaluate the practical applicability and computational cost of the proposed hybrid framework, a performance benchmark was executed. The entire validation database, comprising 800 independent multipath wave propagation profiles, was processed sequentially in a continuous computational loop with graphical rendering deactivated. The benchmark was conducted within a standard Matlab environment using a conventional desktop computer equipped with an Intel Core i7-12700 processor and 16 GB of RAM. The statistical mass test revealed that the average execution time required to complete the entire localization pipeline for a single data profile is exactly 0.51 s.

Although the current unoptimized Matlab prototype serves as an offline research platform, achieving a single-digit second execution time on standard office hardware clearly demonstrates the system’s operational viability. Porting the geometric equations to a compiled language and deploying the neural network layer via optimized inference engines will inherently reduce the processing latency, enabling real-time or near-real-time deployment. Furthermore, in a practical spectrum monitoring scenario, continuous pipeline calculation is highly inefficient. The framework is designed for an event-triggered architecture, where a detection algorithm continuously monitors the spectrum, while a raw multichannel I/Q data stream is saved into a circular memory buffer. Upon signal detection, the complete localization pipeline is invoked exclusively for a narrow time-window extracted from the buffer (e.g., several seconds surrounding the event triggers).

## 4. Discussion

The empirical and theoretical results documented in this study demonstrate that the proposed hybrid localization framework provides a powerful solution to the long-standing challenge of non-cooperative emitter tracking in harsh, NLOS urban environments under strict sparse data constraints. The fundamental synergy of this architecture lies in its ability to bridge the gap between physics-based propagation geometry and data-driven pattern recognition. As evidenced by the comparative analysis, a standalone backward raytracing engine completely collapses when forced to operate on raw directional inputs degraded by multipath correlation and CM-MUSIC subspace leakage, resulting in continuous spatial ambiguities and massive linear displacements. By introducing the convolutional neural network as a high-level semantic filter, the framework effectively decodes the underlying spatial feature embedded within the multi-peak pseudospectrum. The CNN identifies the active monitoring sector and zeroes out peripheral geometric noise and false ray intersections, allowing the core centroid engine to achieve an exceptional operational precision, characterized by a global sample median error of just 9.22 m.

Despite this high average resolution, a critical analysis of the framework’s boundaries reveals distinct operational and physical limitations that must be addressed. The primary algorithmic vulnerability of the system is its high sensitivity to macro-region classification failures within the neural network layer. As analyzed via the confusion matrix when the CNN misclassifies a wavefront realization across the primary eastern and western macro perimeters, the resulting spatial mask suppresses the true physical propagation corridors. This failure forces the centroid computation to execute entirely on isolated, false multipath artifacts, driving the linear displacements immediately to the documented maximum outlier bound of 410.68 m. Furthermore, the methodology exhibits a structural dependence on the availability of a 3D urban model, and its empirical validation was constrained to a single stationary base station intercepting a narrow-band PMR frequency. Under operational scenarios featuring highly dynamic mobile receivers or multi-frequency wideband frequency-hopping emitters, the spatial phase relationships within the covariance matrix would experience rapid fluctuations, potentially increasing the structural error rate of the raw feature inputs.

The comparative bound analysis executed in the theoretical upper-performance scenario yields a vital architectural conclusion regarding these algorithmic limitations. When the framework is insulated from semantic sector masking errors, the baseline combination of the CM-MUSIC estimator, backward raytracing, and centroid extraction delivers a global mean localization accuracy of 9.30 m, with 50% of all evaluation samples tightly packed within the 4.52 m interquartile bracket. The minor residual outliers observed within this ideal bound, which peak at a maximum of 34.29 m, are exclusively driven by the deterministic abstractions of the 3D model, where complex, multi-wing building geometries were simplified into cuboids. Consequently, this insight proves that the physical wave-tracking and centroid localization pipeline is already highly optimized and structurally complete. This explicitly isolates the deep learning sector classifier as the primary performance bottleneck of the entire framework. Therefore, subsequent code optimization efforts should focus entirely on enhancing the classification accuracy and multi-class resilience of the CNN layer, rather than modifying the underlying geometric raytracing equations.

From a practical and operational perspective, the developed hybrid framework offers substantial logistical advantages for modern electronic reconnaissance and civilian spectrum regulatory authorities, such as national radio monitoring agencies. Conventional high-precision urban localization frameworks typically rely on dense, highly cooperative sensor networks or massive, expensive multi-site direction-finding infrastructure that require extensive spatial synchronization and high data link bandwidths. In sharp contrast, the system developed in this study achieves sub-ten-meter median accuracy using highly fragmented empirical data captured from a single, resource-constrained monitoring platform. By deploying a low-cost, 5-channel coherent SDR architecture integrated onto a single mobile vehicle, operators can effectively execute autonomous passive tracking of non-cooperative emitters entirely from a stationary base position. The framework’s ability to successfully exploit multipath reflections as virtual source pathways, rather than treating them as destructive interference, eliminates the requirement for a direct LOS path providing an efficient, highly scalable, and hardware-preserving methodology for advanced urban spectrum enforcement.

## 5. Conclusions

This paper introduced a novel hybrid localization framework designed for non-cooperative single-site emitter tracking in dense urban environments under NLOS conditions and sparse empirical data availability. The developed architecture achieves robust tracking capabilities by combining advanced signal processing, machine learning pattern recognition, and physics-based wave propagation models. Through the implementation of a geometry-aware CM-MUSIC algorithm, spatial phase dependencies were successfully preserved on a resource-constrained 5-element UCA without physical aperture loss. The integration of a CNN acting as a macro-sector semantic mask effectively isolated the true physical propagation corridors, eliminating the spatial ambiguities and false ray intersections that typically cause standalone geometric engines to fail.

The empirical validity and resilience of the complete framework were proven under strict blind-test operational conditions using FM transmissions captured via a low-cost SDR radio platform mounted on a single stationary vehicle. The experimental results demonstrated high tracking precision, yielding an exceptional global sample median localization error of just 9.22 m and an absolute minimum error bound of 2.05 m. While systemic cross-macro-region classification errors within the neural network layer skewed the global mean error to 55.52 m by generating high-magnitude spatial outliers, the baseline geometric engine demonstrated extraordinary potential. The ideal sector-classification scenario analysis confirmed that under ideal sector masking conditions, the combined CM-MUSIC and backward raytracing pipeline converges to a global mean localization error of 9.3 m, with half of all validation points tightly bound with a 4.52 m interquartile range.

Nevertheless, the proposed framework still suffers from outlier results reaching up to 410.68 m linear error. Although this is not a typical value, it leads to the conclusion that treating the CNN result as a hard sector masking might not be the most efficient solution. Further research on this subject, including treating class probabilities as weights, must be conducted, which would probably limit outlier results that currently appear in the proposed framework.

The significant localization accuracy achieved from a single mobile monitoring station establishes a highly scalable and cost-effective framework for modern electronic reconnaissance and civilian radio frequency regulatory enforcement. In future research, the operational capabilities of this hybrid framework will be extended beyond stationary intercepts. Future work will focus on adapting the underlying signal processing models to track dynamic mobile emitters in real-time, as well as validating the system’s tracking resilience for wideband emissions and complex frequency-hopping wave profiles. It is also necessary to repeat current research with more measurement positions among each sector during the CNN training session and conduct research for unknown emitter positions during the test session. This would help to ensure frameworks’ robustness for completely unknown signal profiles, which is the core assumption of our framework.

## Figures and Tables

**Figure 1:**
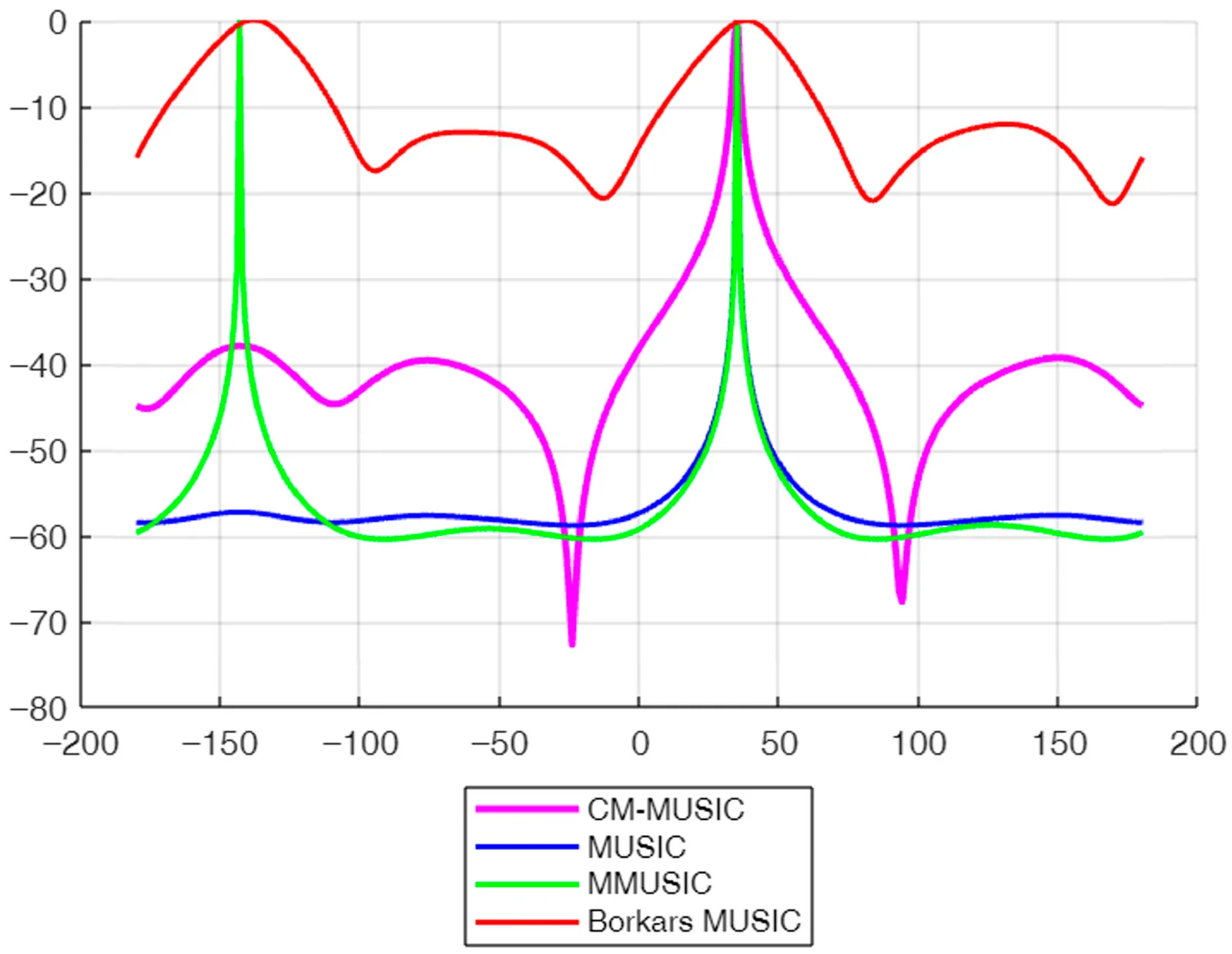
Simulation Scenario 1 Results.

**Figure 2:**
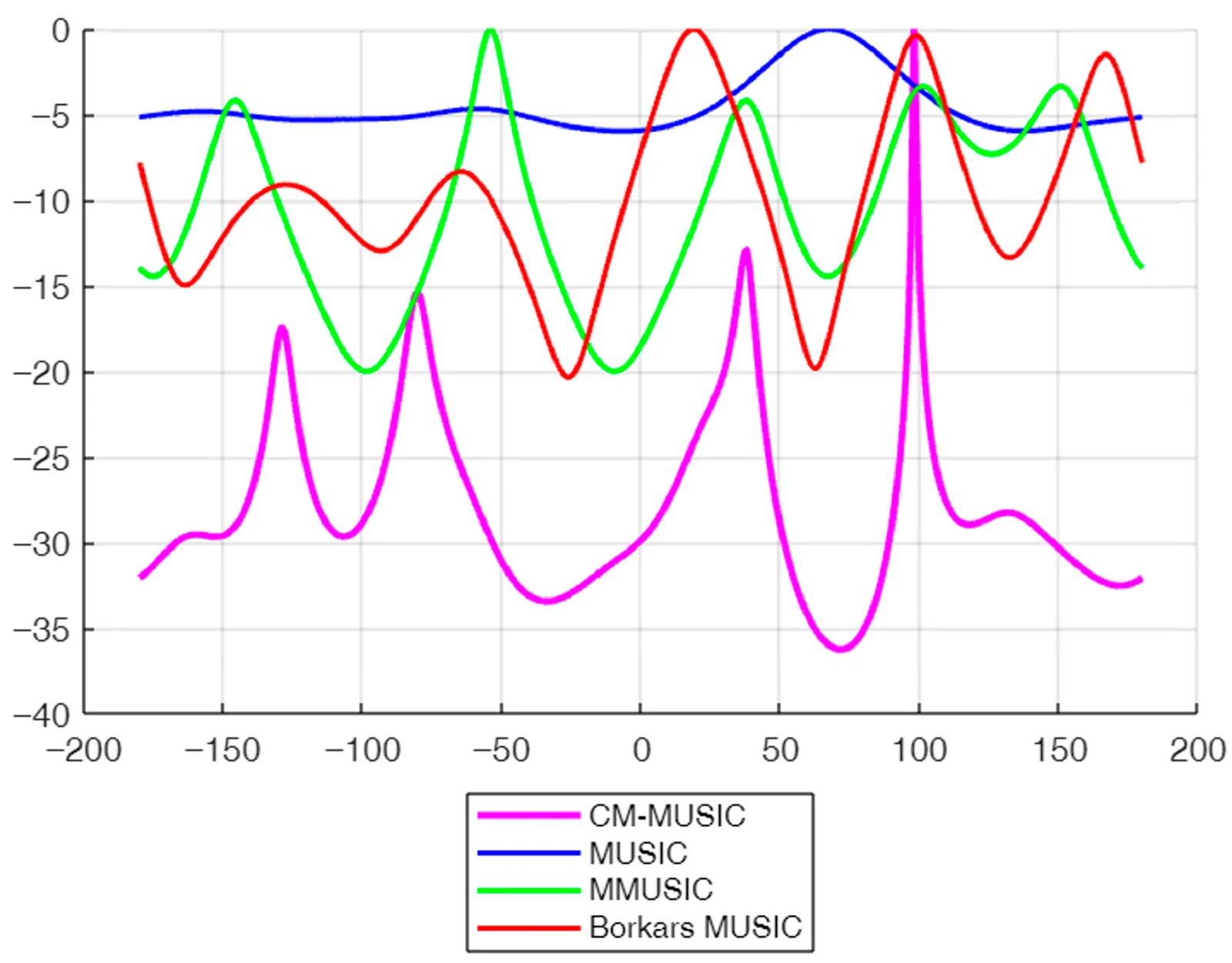
Simulation Scenario 2 Results.

**Figure 3:**
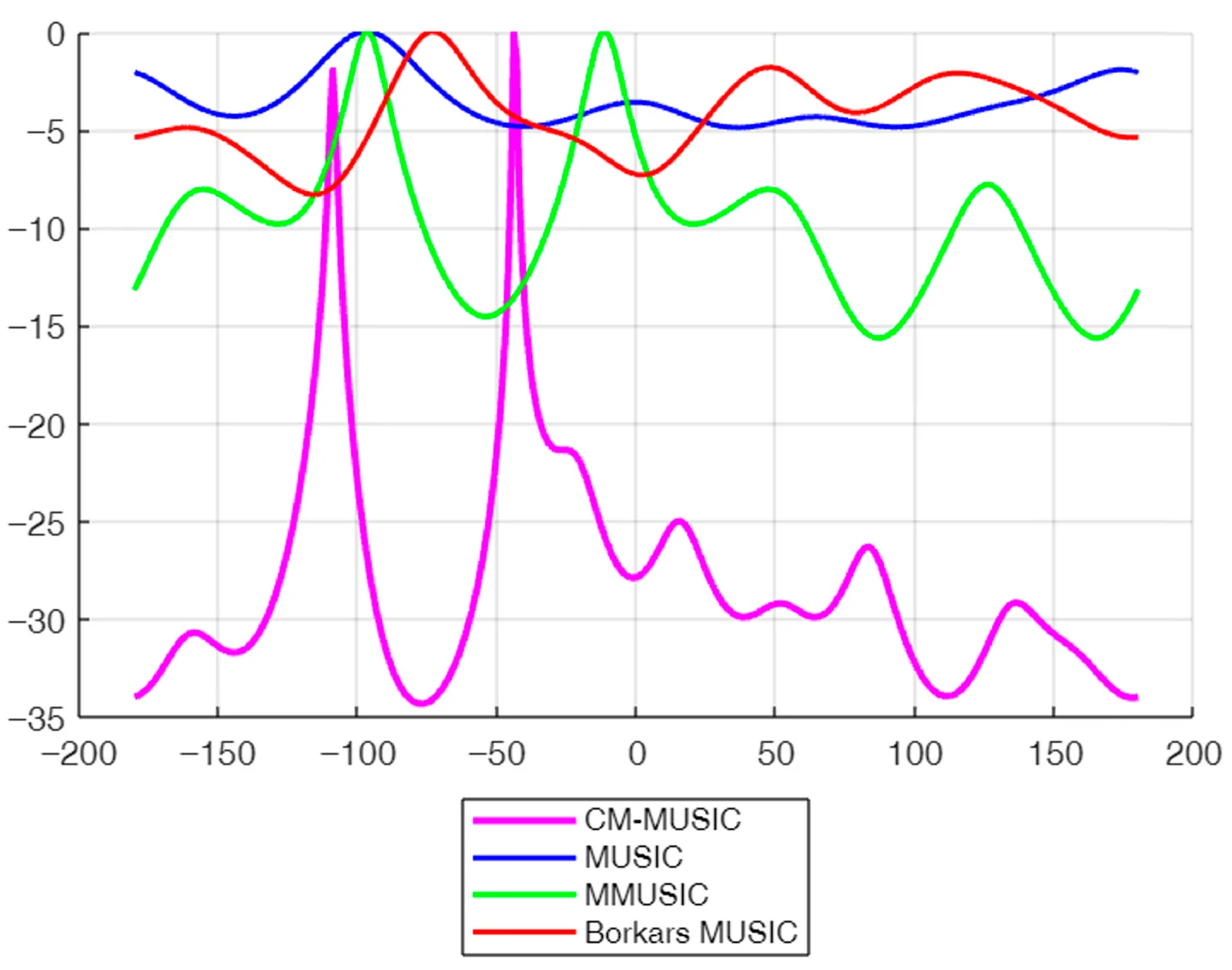
Simulation Scenario 3 Results.

**Figure 4:**
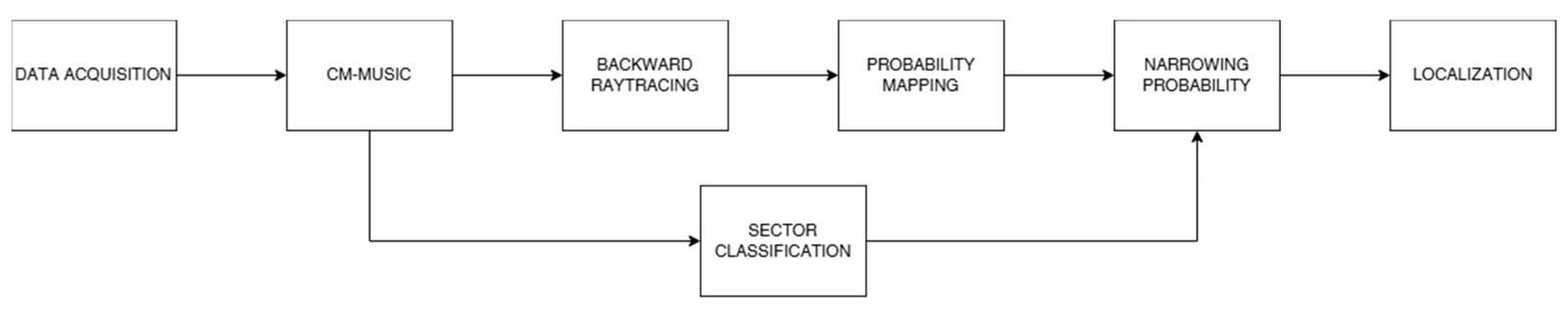
Framework Pipeline.

**Figure 5:**
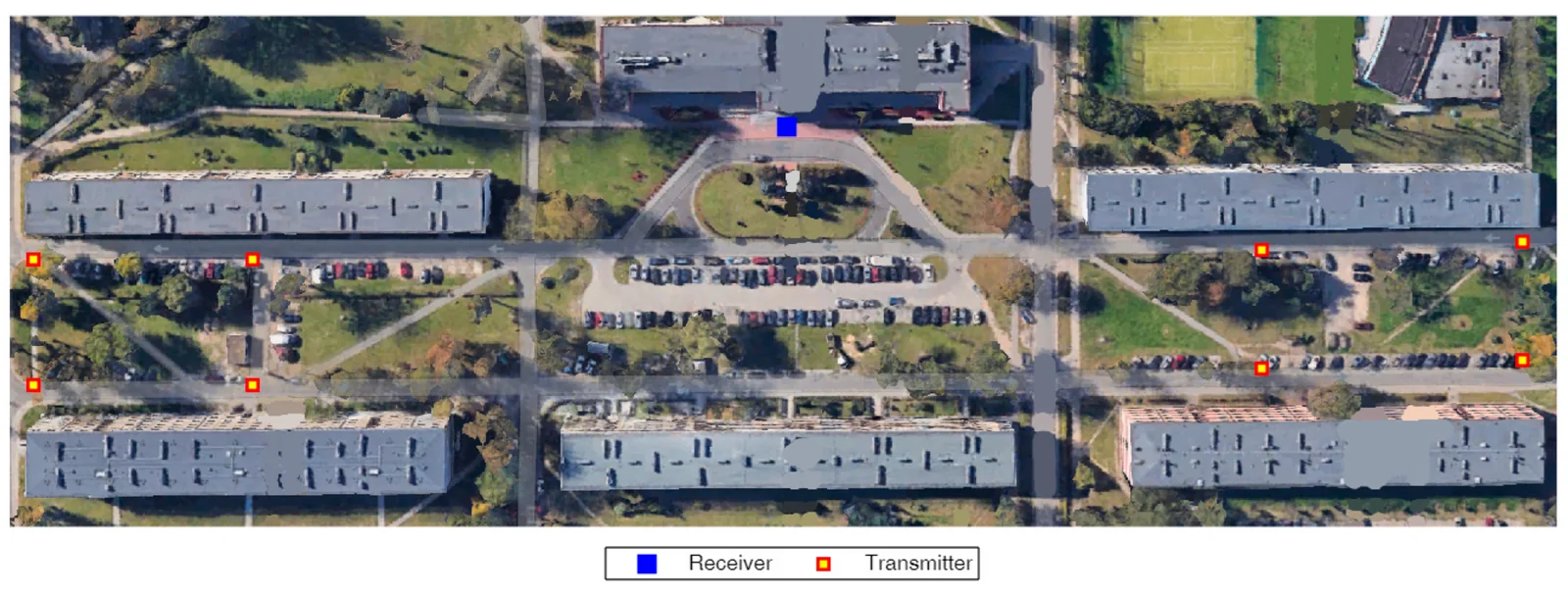
Map of the measurement.

**Figure 6:**
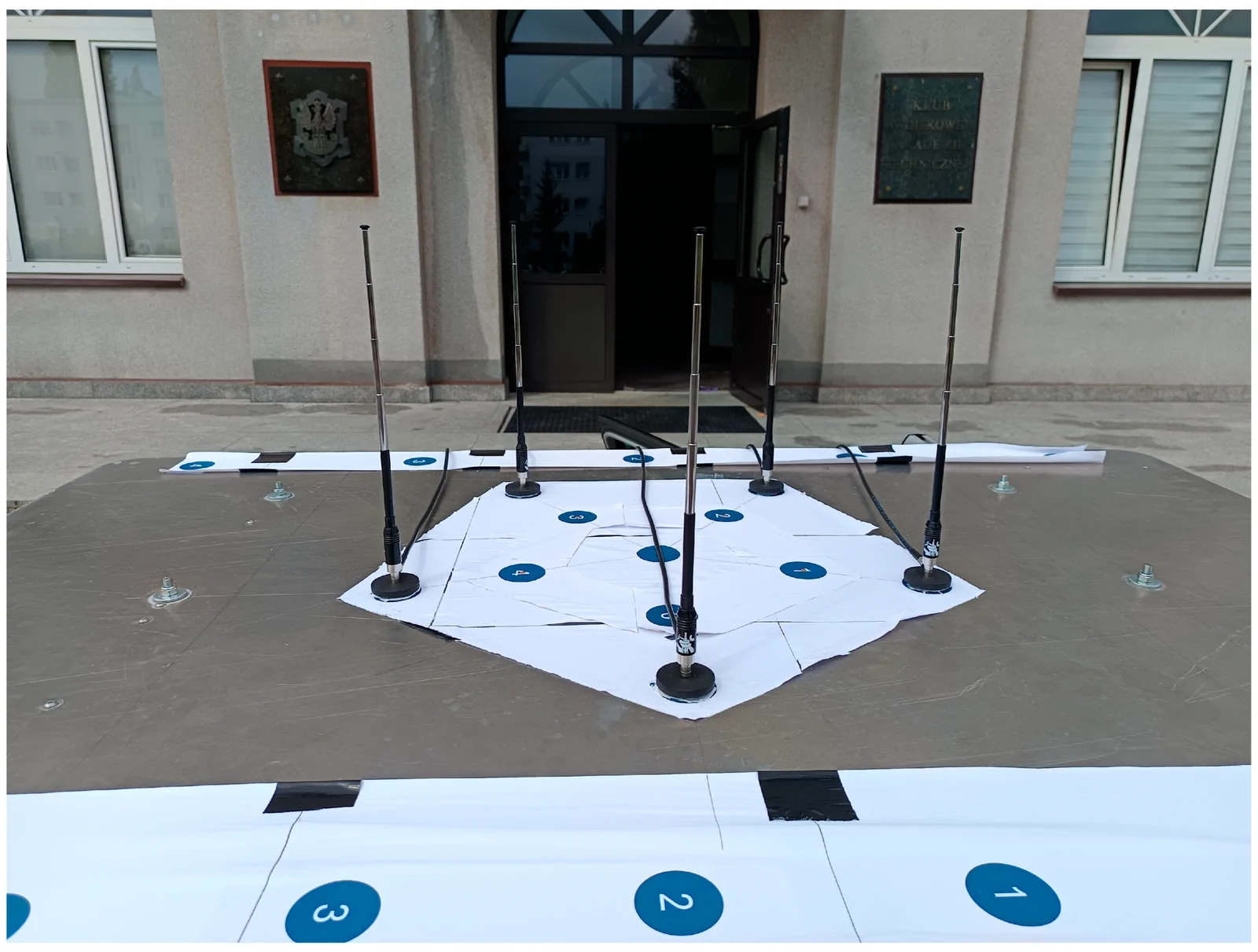
Antennas mounted on the measurement vehicle.

**Figure 7:**
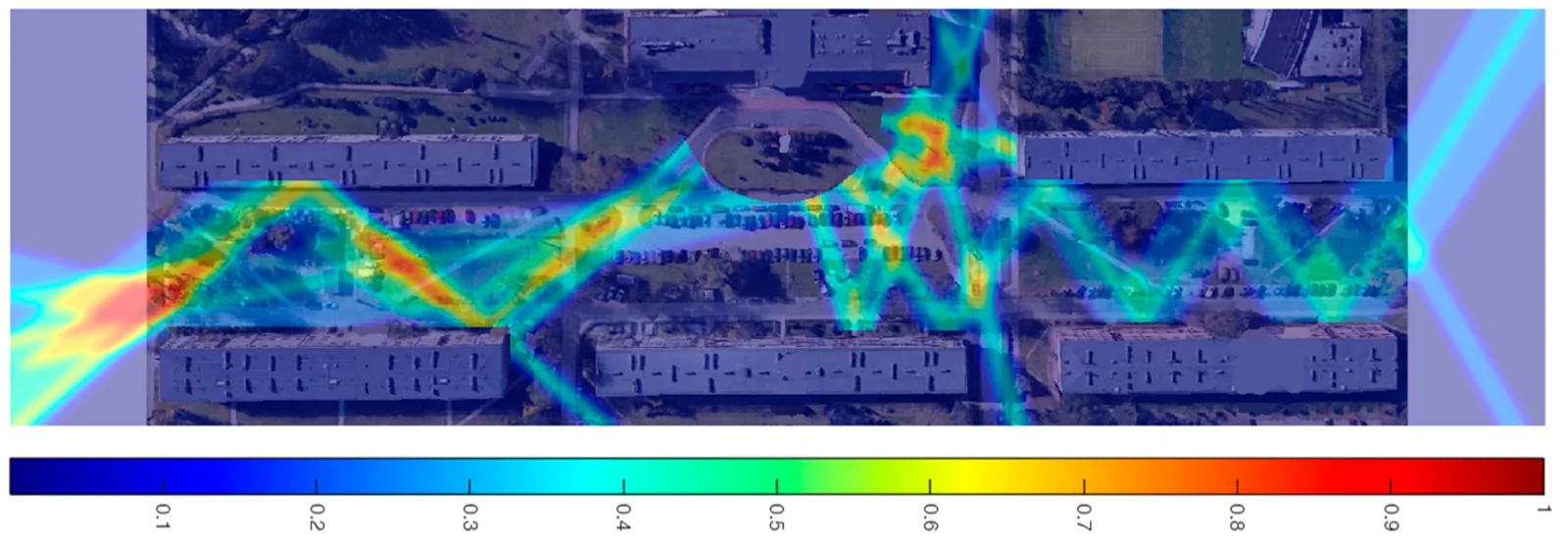
Heatmap visualization of the spatial likelihood.

**Figure 8:**
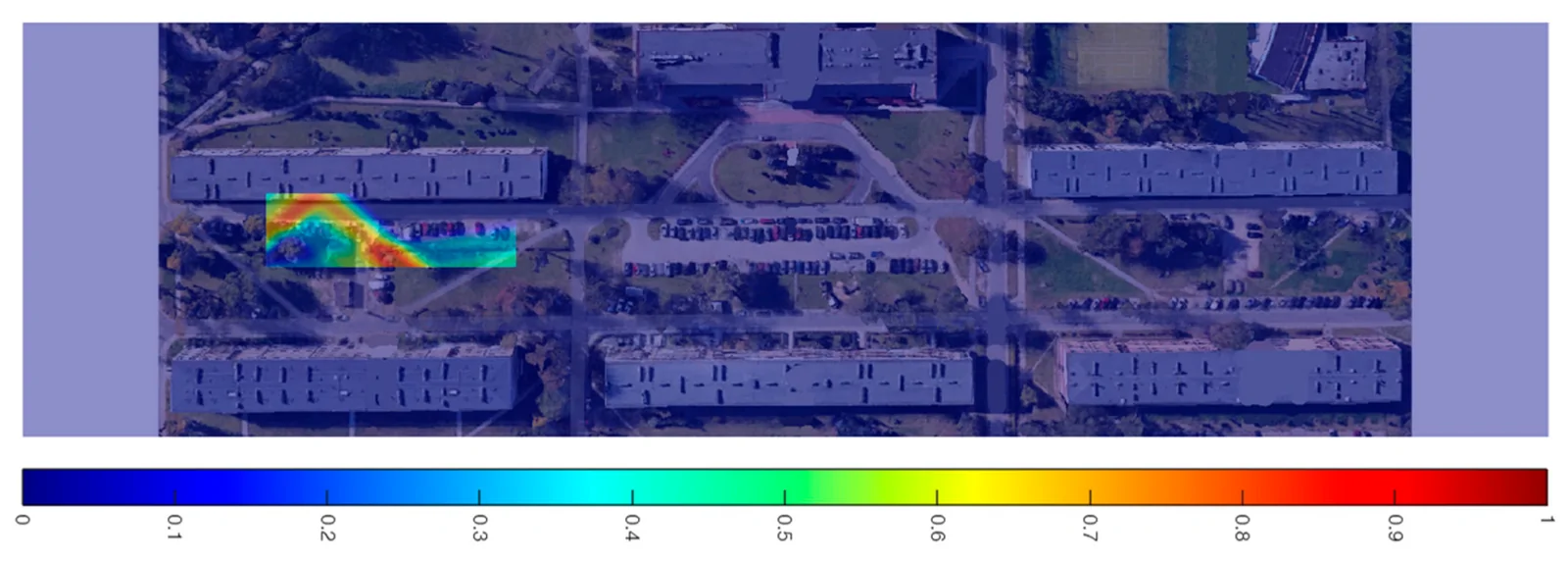
Heatmap visualization of the spatial likelihood map after sector filtering.

**Figure 9:**
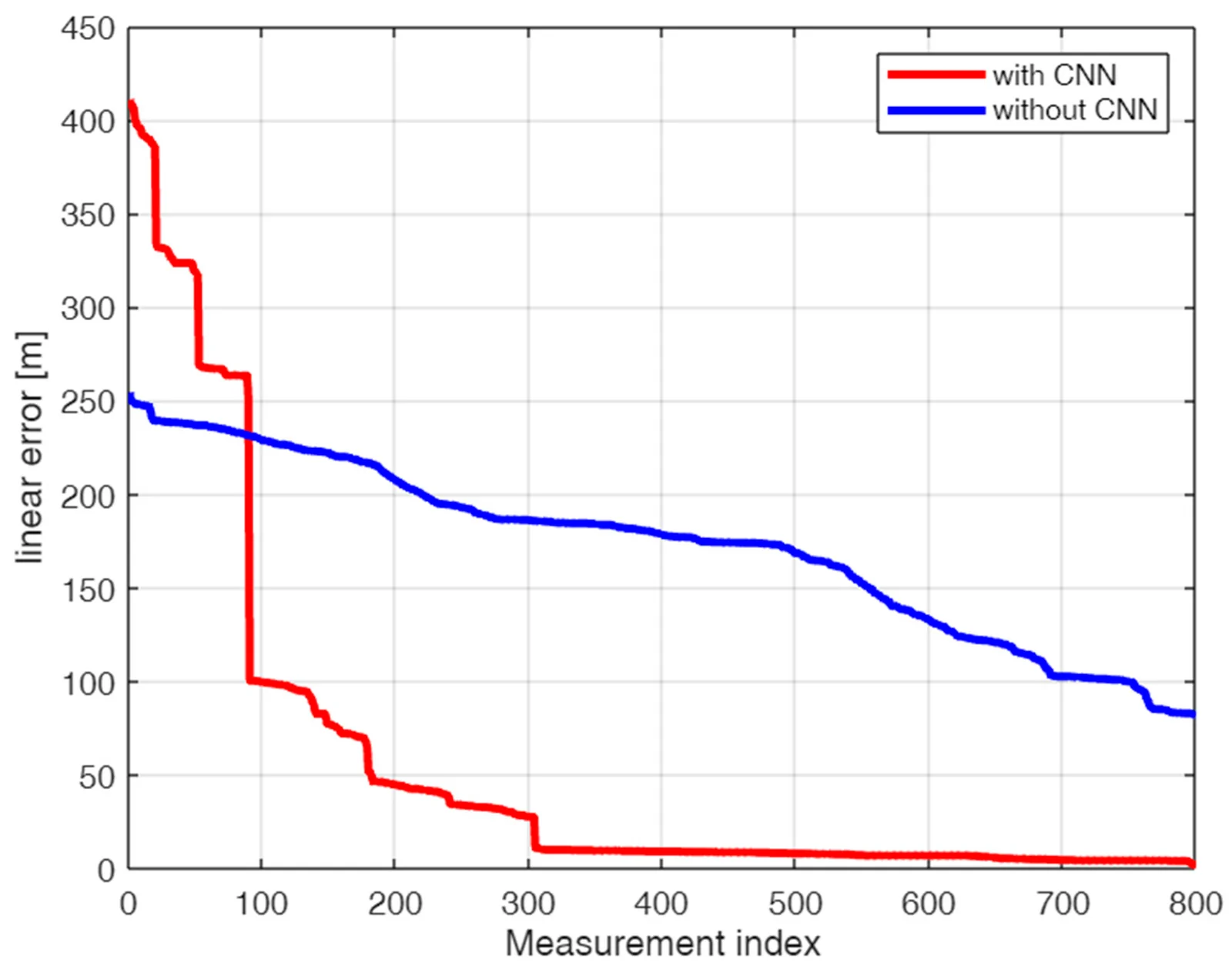
Localization linear error for the proposed framework (with CNN) compared to geometric method (without CNN).

**Figure 10:**
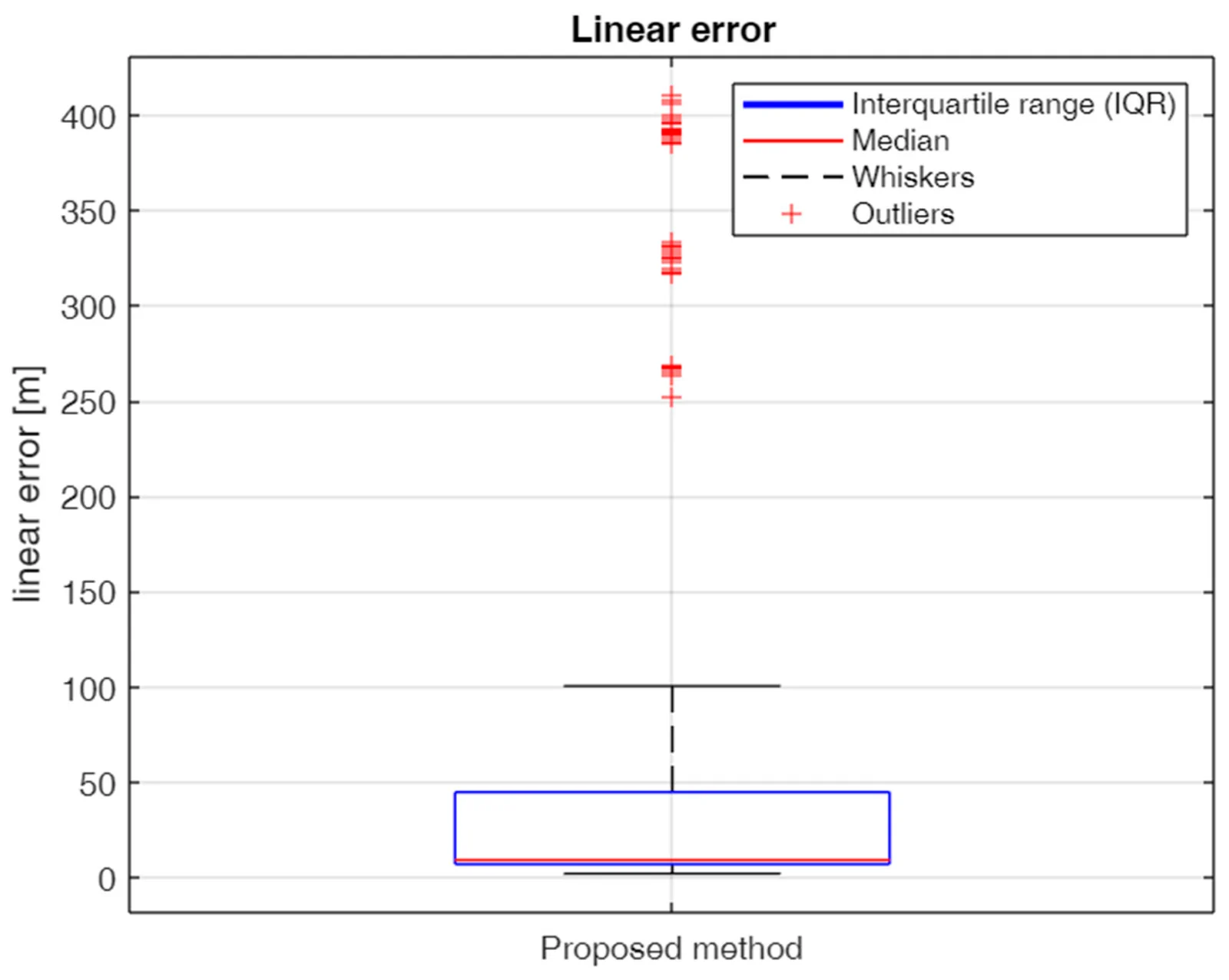
Boxplot distribution of the linear error of the framework.

**Figure 11:**
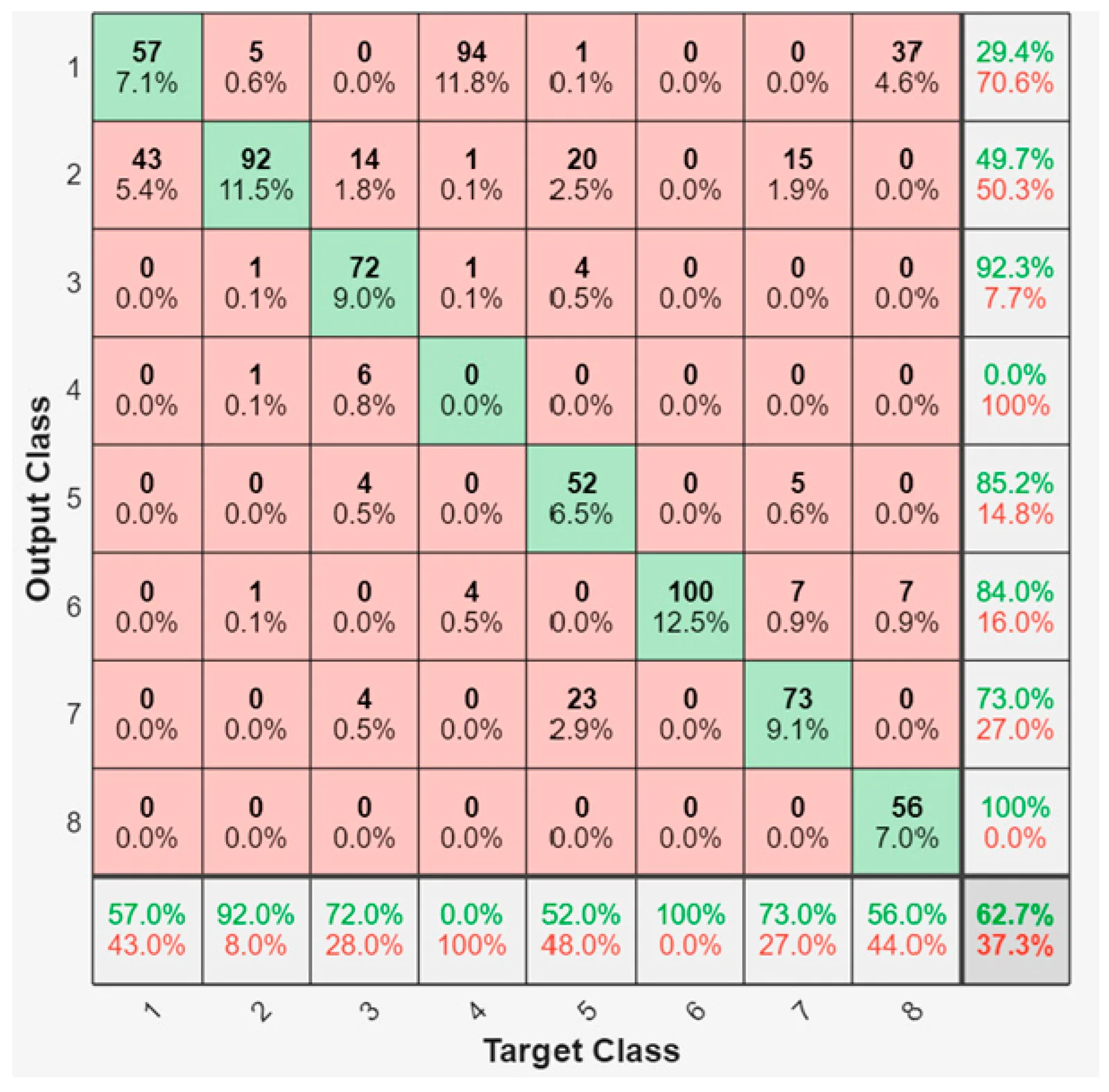
Created CNN Confusion Matrix, including accuracy results (green numbers) and—Green cells—correctly classified samples—Pink cells—misclassification—Grey cells—precision (top, green) and false (bottom red).

**Figure 12:**
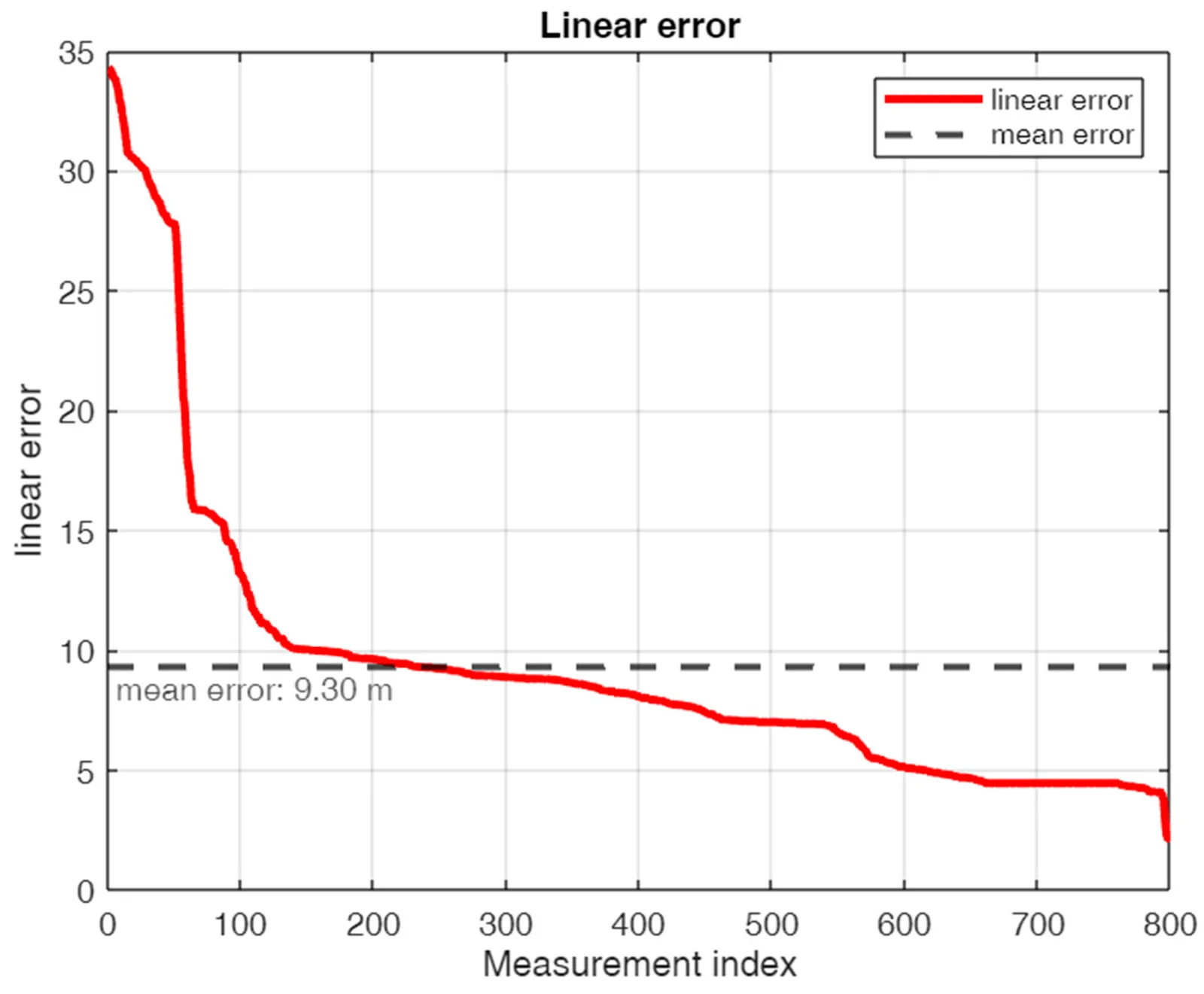
Linear Error for Ideal Sector-Classification Scenario.

**Figure 13:**
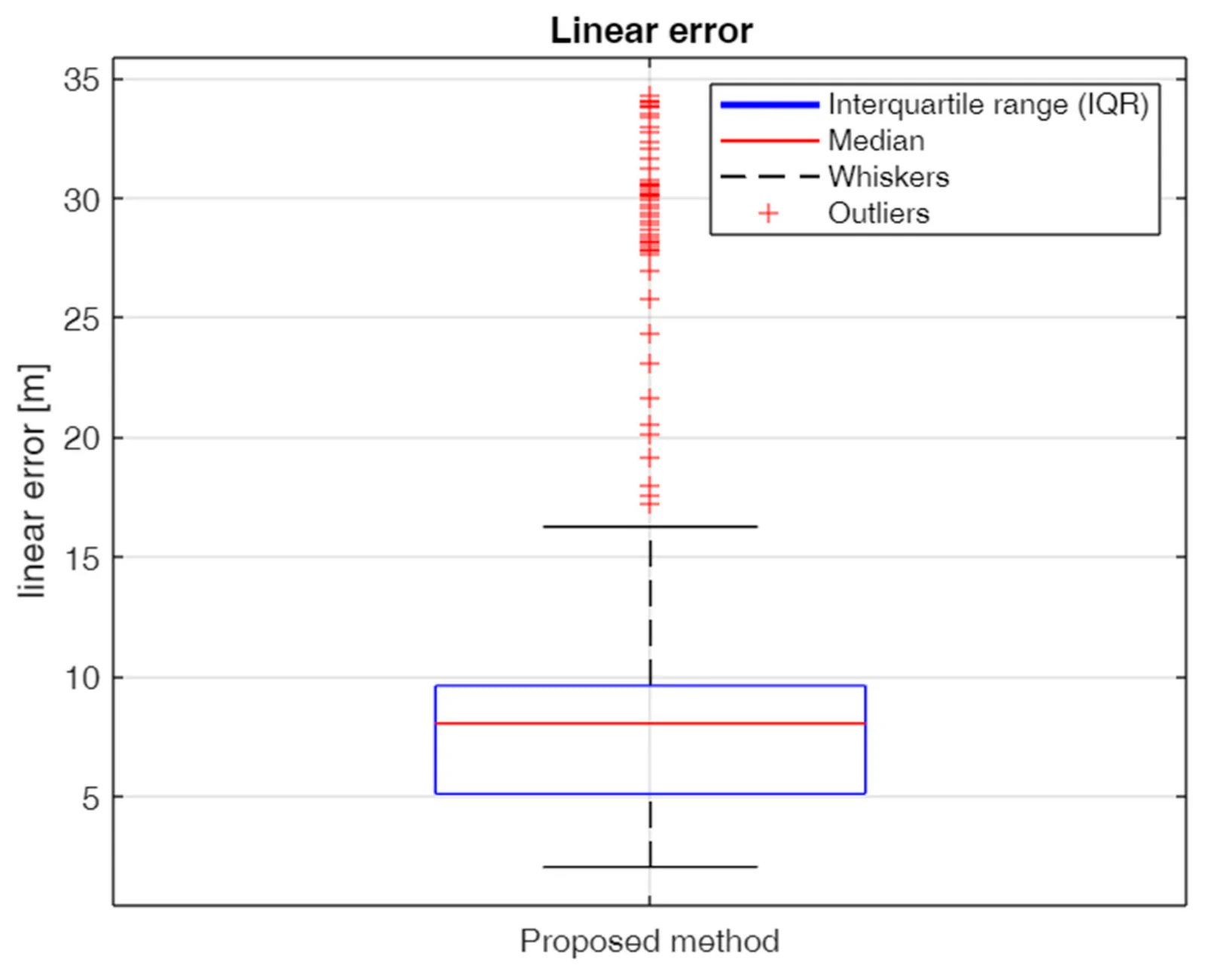
Boxplot of a Linear Error for Ideal Sector-Classification Scenario.

**Table 1:** Simulation Parameters.

Parameter	Value
Array Geometry	5-element UCA
Radius	26.3 cm
Operating Frequency	446.15625 MHz
Signal Modulation	FM (1 kHz tone)
Snapshot Volume	100,000 snapshots
SNR	10 dB

**Table 2:** CNN Structure.

Layer Number	Layer Type	Output Shape/Hyperparameters	Dropout Rate
1	Image Input	2 × 765 × 1	-
2	2D Convolution	128 filters, Kernel: [2 × 5]	-
3	Batch Normalization	-	-
4	ReLU	-	-
5	Flatten	1D Vector	-
6	Fully Connected	4096 neurons	-
7	ReLU	-	-
8	Dropout	Probability: 0.60	0.60
9	Fully Connected	2048 neurons	-
10	ReLU	-	-
11	Dropout	Probability: 0.55	0.55
12	Fully Connected	1024 neurons	-
13	ReLU	-	-
14	Dropout	Probability: 0.5	0.50
15	Fully Connected	512 neurons	-
16	ReLU	-	-
17	Dropout	Probability: 0.45	0.45
18	Fully Connected	128 neurons	-
19	ReLU	-	-
20	Dropout	Probability: 0.35	0.35
21	Fully Connected	64 neurons	-
22	ReLU	-	-
23	Dropout	Probability: 0.30	0.30
24	Fully Connected	8 neurons	-
25	Softmax	-	-
26	Classification Output	Cross-Entropy Loss	-

## Data Availability

The simulation code and data used in this study are available from the corresponding author upon reasonable request.

## Abbreviations

The following abbreviations are used in this manuscript:

UCA	Uniform Circular Array
CNN	Convolutional Neural Network
SDR	Software Defined Radio
IoT	Internet of Things
LOS	Line-Of-Sight
NLOS	Non-Line-Of-Sight
DOA	Direction of Arrival
ULA	Uniform Linear Array
MUSIC	Multiple Signal Classification
AWGN	Additive White Gaussian Noise
EVD	Eigenvalue Decomposition
MMUSIC	Modified MUSIC
SVD	Singular Value Decomposition
CM-MUSIC	Circulant Modified MUSIC
PMR	Private Mobile Radio
FM	Frequency Modulated
SNR	Signal to Noise Ratio
ReLU	Rectified Linear Unit
Adam	Adaptive Moment Estimation
IQR	Interquartile Range
